# In vivo study of a novel 3D-printed motion-preservation artificial cervical corpectomy construct: short-term imaging and biocompatibility evaluations in a goat model

**DOI:** 10.1186/s13018-024-04786-w

**Published:** 2024-05-28

**Authors:** Jian Wang, Bing Meng, Xinli Wang, Wei Lei, Xiong Zhao

**Affiliations:** 1 Department of Orthopaedics, Xijing Hospital, Air Force Military Medical University, Xi’an, 710032 Shaanxi Province China; 2 Department of Orthopaedics, Affiliated Hospital of NCO School of Army Medical University, Shijiazhuang, 050047 Hebei Province China

**Keywords:** Nonfusion technology, Motion-preservation device, 3D printing technology, Porous metal, Spinal stability

## Abstract

**Background:**

Nonfusion technologies, such as motion-preservation devices, have begun a new era of treatment options in spine surgery. Motion-preservation approaches mainly include total disc replacement for anterior cervical discectomy and fusion. However, for multisegment fusion, such as anterior cervical corpectomy and fusion, the options are more limited. Therefore, we designed a novel 3D-printed motion-preservation artificial cervical corpectomy construct (ACCC) for multisegment fusion. The aim of this study was to explore the feasibility of ACCC in a goat model.

**Methods:**

Goats were treated with anterior C3 corpectomy and ACCC implantation and randomly divided into two groups evaluated at 3 or 6 months. Radiography, 3D CT reconstruction and MRI evaluations were performed. Biocompatibility was evaluated using micro-CT and histology.

**Results:**

Postoperatively, all goats were in good condition, with free neck movement. Implant positioning was optimal. The relationship between facet joints was stable. The range of motion of the C2-C4 segments during flexion–extension at 3 and 6 months postoperatively was 7.8° and 7.3°, respectively. The implants were wrapped by new bone tissue, which had grown into the porous structure. Cartilage tissue, ossification centres, new blood vessels, and bone mineralization were observed at the porous metal vertebrae-bone interface and in the metal pores.

**Conclusions:**

The ACCC provided stabilization while preserving the motion of the functional spinal unit and promoting bone regeneration and vascularization. In this study, the ACCC was used for anterior cervical corpectomy and fusion (ACCF) in a goat model. We hope that this study will propel further research of motion-preservation devices.

## Introduction

Various pathologies, such as fractures, tumours, or infections, can affect the vertebral body, leading to instability, pain, and spinal deformity [1]. Spinal fusion, an invaluable tool in the spine surgeon’s armamentarium since it was first reported by Hibbs and Albee in 1911 [2, 3], remains the gold standard surgical treatment for these spinal pathologies [4]. However, the drawbacks to fusion, including acceleration of adjacent segment disease (ASD), alteration of spinal biomechanics, pseudarthrosis, and bone graft donor site pain, have increased [5], and these issues have led the surgical community to explore novel approaches.

Nonfusion technologies, such as motion-preservation devices, have begun a new era of treatment options [6]. These devices are designed with the intent to provide stabilization while preserving the motion of the functional spinal unit [6]. Given their advantages, which include restoring spinal alignment, reducing adjacent segment degeneration, decompressing neural elements while preserving functional motion, and eliminating the need for bone grafting, nonfusion technologies have been suggested as an alternative to fusion and may become the new gold standard [6]. At present, the motion-preservation devices used in the cervical spine mainly include total disc replacement devices for anterior cervical discectomy and fusion (ACDF) [7]. However, for multisegment fusion, such as anterior cervical corpectomy and fusion (ACCF), there are fewer options for cervical motion preservation surgery [7].

In recent years, 3D printing technologies have rapidly increased in popularity and are widely used in the field of medicine [8]. Therefore, we designed a method for constructing a novel porous titanium alloy motion-preservation artificial cervical corpectomy construct (ACCC) using 3D printing technology. Prior to implantation, such medical devices should be subjected to rigorous testing to ensure their efficacy and safety [9]. Here, imaging and biocompatibility evaluations were performed for the first time to test the ACCC in a goat model.

## Materials and methods

### Study design

This study was approved by the Animal Care Committee of our institution (permit number: 20160606). The Animal Care Committee of our institution waived the informed consent to be obtained from the experimental subject because this study was animal study design.

Sixteen adult Chinese white goats (age, 18.7 ± 1.8 months; weight, 37.6 ± 2.8 kg) were selected from the Animal Centre of Xijing Hospital, Fourth Military Medical University. Each sheep underwent anterior C3 corpectomy and ACCC implantation. All goats were randomly divided into 2 groups (n = 8 per group): group A (goats observed for 3 months) and group B (goats observed for 6 months). Imaging evaluations were performed in all of the goats by radiography, 3D CT reconstruction and MRI. Then, four goats were randomly selected from each group for the biocompatibility evaluation, consisting of micro-CT and histology. The other four goats in groups A and B were used for subsequent studies.

### Design and fabrication of the ACCC

The design of the ACCC stemmed from the concept of nonfusion technology and was intended to provide stabilization while preserving the motion of the functional spinal unit [6]. The shape and size of the ACCC device were designed based on 3D CT reconstruction of the cervical spine. A porous structure was built on the surface of the artificial vertebral body to facilitate bone ingrowth. We used 3D printing technology during the manufacturing process to successively melt titanium alloy powder (Ti6Al4V) layer by layer according to a computer-aided design model.

### Surface characterization of the ACCC

The characteristics of the surface morphology of the ACCC were detected using scanning electron microscopy (SEM). The samples were mounted on an SEM sample holder, and 6 images of the microstructure were randomly acquired. The average dimension of the pores was calculated based on the microstructural images.

### Imaging evaluation

X-ray radiography was performed at 1 day, 3 months and 6 months after surgery to evaluate the position and subsidence of the implants. 3D CT reconstruction and MRI were performed at 3 months and 6 months after surgery to evaluate the position of the implants, the relationship between facet joints, the range of motion (ROM) of the operative level under flexion–extension, details of the spinal cord, nerve roots and adjacent intervertebral discs, and adverse events, such as heterotopic ossification. Due to difficulty in positioning, the ROM was not measured at the operative level during lateral bending to the right/left or axial rotation to the right/left. Preoperative ROM of the C2-C4 segments was used as the control group. All equipment was manufactured by Siemens (Germany).

### Micro-CT analysis

The C2-C4 segments of the cervical spine were harvested and placed in a sample holder for micro-CT, which was performed using a micro-CT machine (YXLON Y. CT, Germany). The X-ray source voltage was set at 80 kV, and the beam current was set at 500 μA using filtered Bremsstrahlung radiation. The micro-CT images were reconstructed using VG Studio MAX software. Based on the above data, the percentage of bone volume out of the pore volume (BV/PV) from each group was measured and statistically analyzed.

### Histological analysis

After micro-CT analysis, the C2-C4 specimens were fixed in 4% paraformaldehyde for 1 month, dehydrated through a series of graded ethanol solutions (70%, 80%, 90%, 95% and 100%) and then embedded in methyl methacrylate. Thin slices (approximately 100 μm in thickness) were prepared from the specimens using a modified interlocked diamond saw (LeicaLA 1600, Germany) and a polishing machine (Ruifeng, Xi’an, China). Then, the slices were stained with 1.2% trinitrophenol and 1% acid fuchsin. Bone formation and bone ingrowth were observed with an automatic light microscope (Leica LA Microsystems, Bensheim, Germany) and an image acquisition system (Penguin 600CL, USA).

### Statistical analysis

Data analysis was carried out using GraphPad Prism 5. Statistical analysis was carried out using one-way ANOVA followed by Bonferroni’s multiple comparison tests. Data are presented as the mean ± standard deviation for each group. A *P* value less than 0.05 was considered statistically significant.

## Results

### Characterization of ACCC

**Fig. 1:**
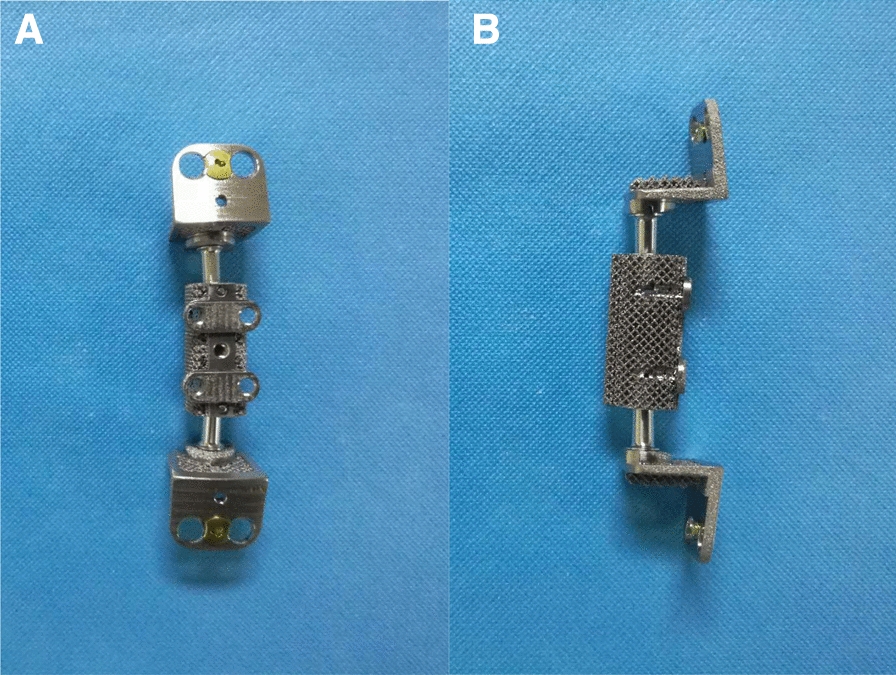
**a** Front view of the ACCC. **b** Side view of the ACCC

**Fig. 2:**
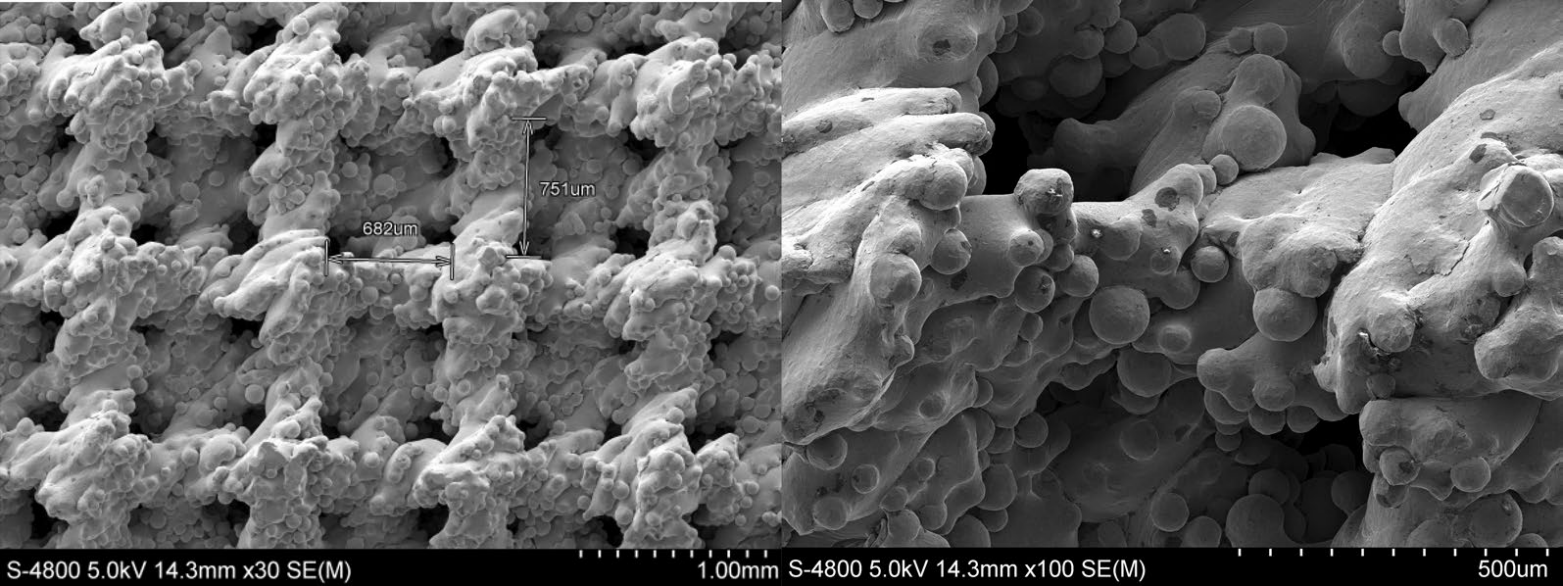
SEM images of the ACCC surface microstructure and pore size

Figure 1 shows the implant product. Figure 2 shows an SEM image of the ACCC surface microstructure and pore size. The surface was rough, with a structure resembling that of trabecular bone.

### Establishment of a goat animal model and health status of goats postoperatively

**Fig. 3:**
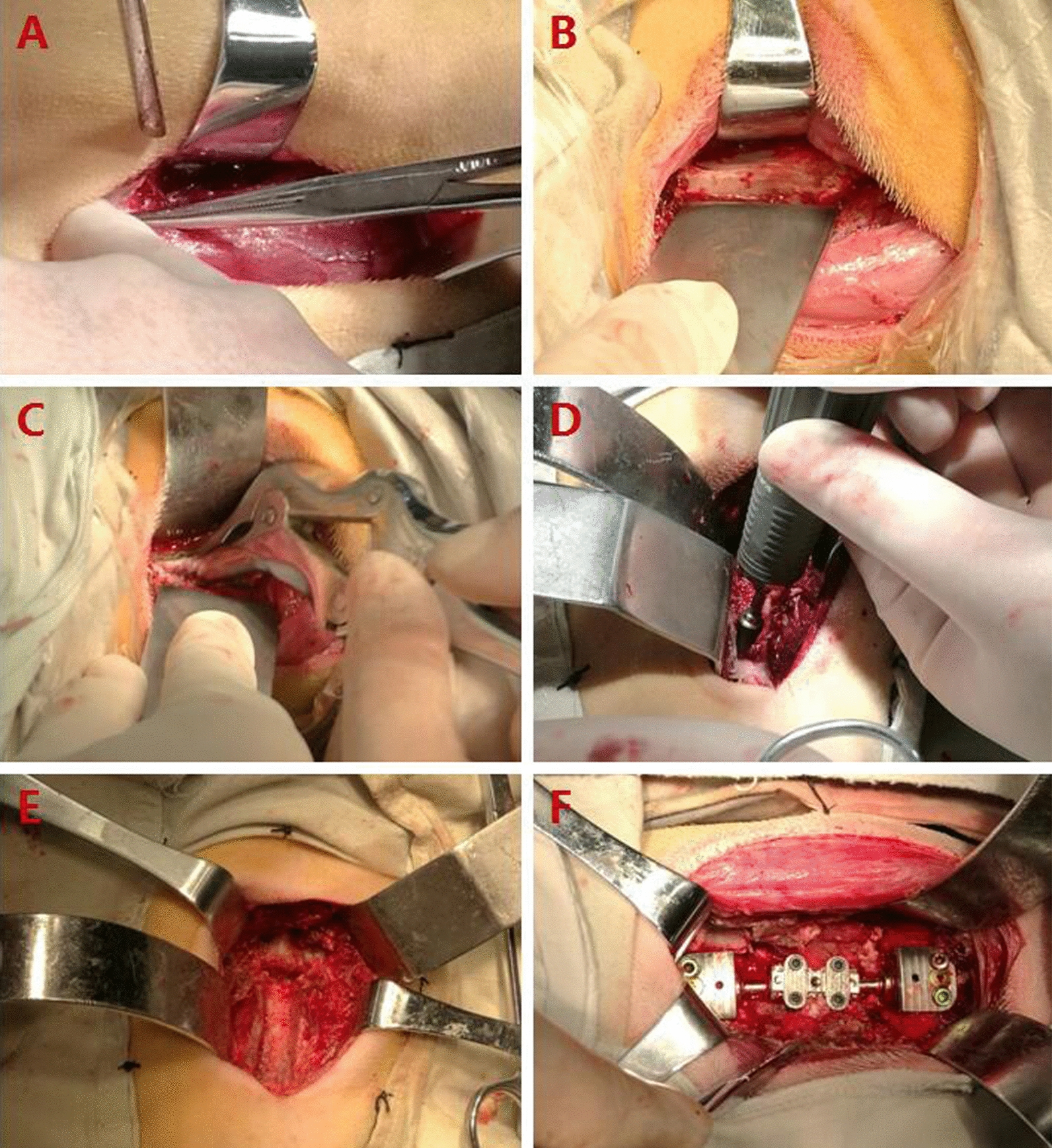
Intraoperative photographs. **A**, A section cut through the skin and subcutaneous tissue. **B**, Exposure of the C3 vertebra. **C,** **D** and **E** C3 corpectomy. **F** ACCC implantation

All 16 goats underwent anterior C3 corpectomy and ACCC implantation (Fig. 3). Throughout the observation period (3 or 6 months after the operation), all goats were in good health with free neck movement and no death or paralysis.

### Radiological evaluation

**Fig. 4:**
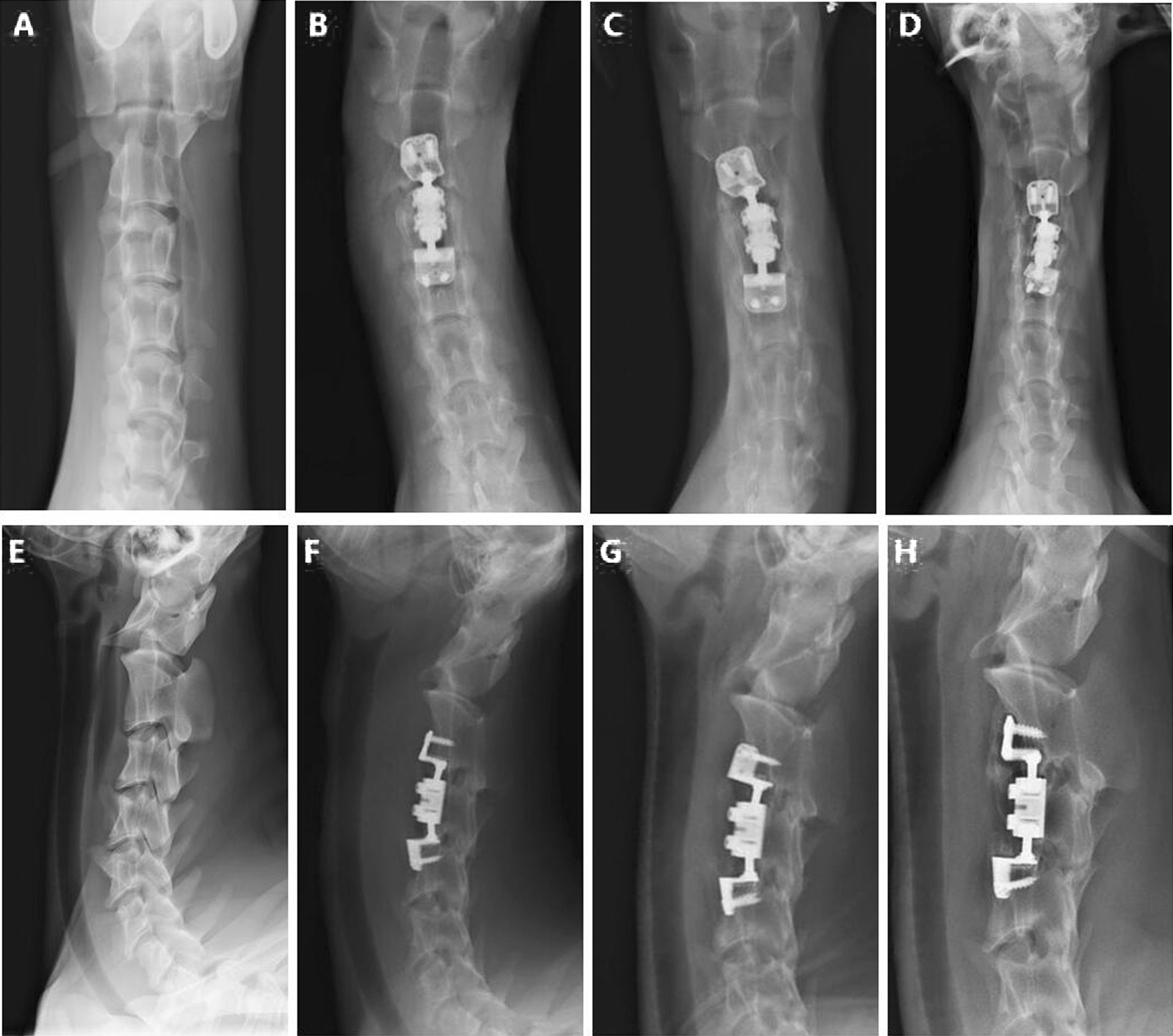
**1** Frontal X-ray film. **A**, pre-operation. **B**, Postoperative day 1. **C**, 3months after surgery. **D**, 6months after surgery. **2** Lateral X-ray film. **E**, pre-operation. **F**, Postoperative day 1. **G**, 3 months after surgery. **H**, 6 months after surgery

X-ray examination suggested that the position of the implant was optimal, and no implant fracture, loosing or implant subsidence was observed (Fig. 4).

**Fig. 5:**
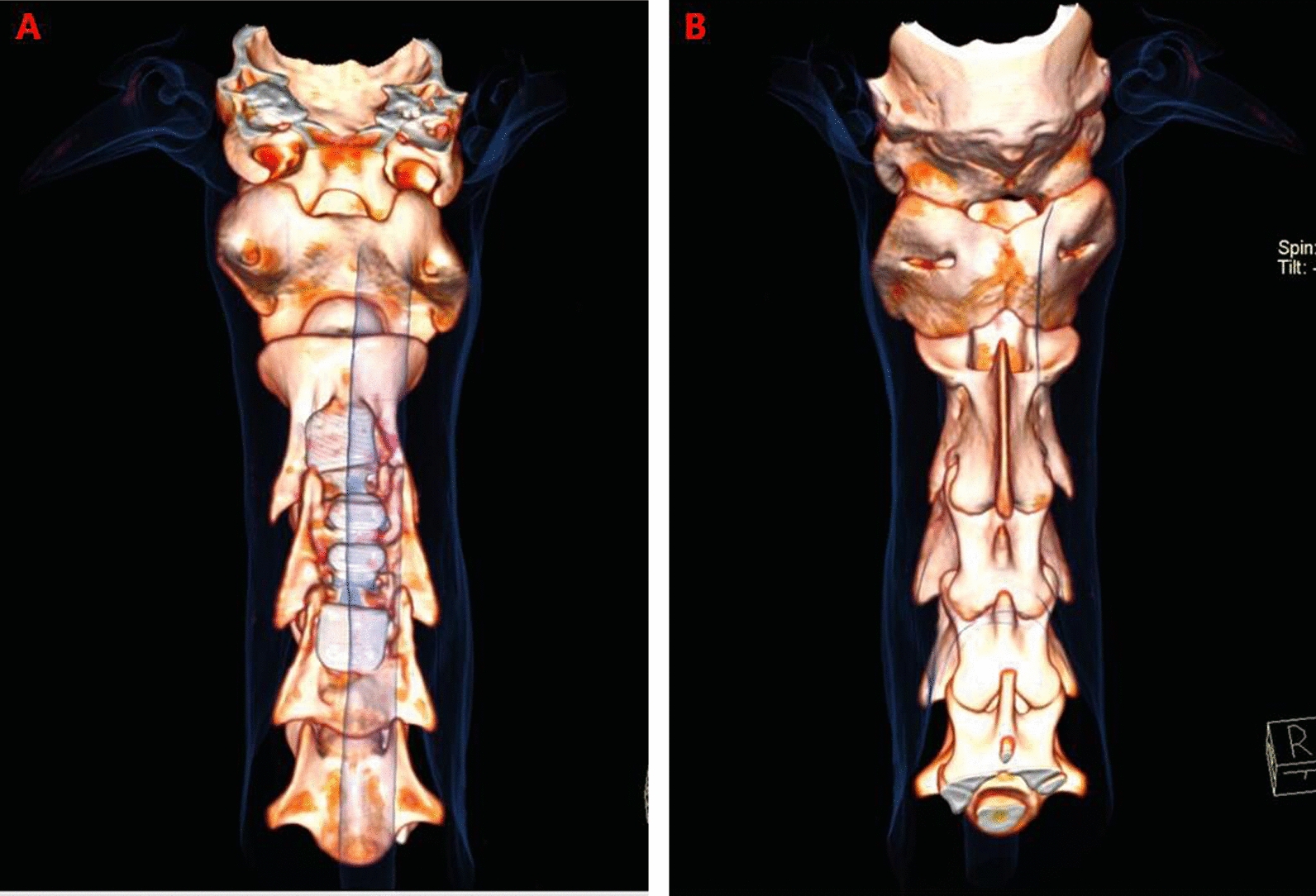
3D CT reconstruction 3 months after surgery. **A**, Front view. **B**, Back view

**Fig. 6:**
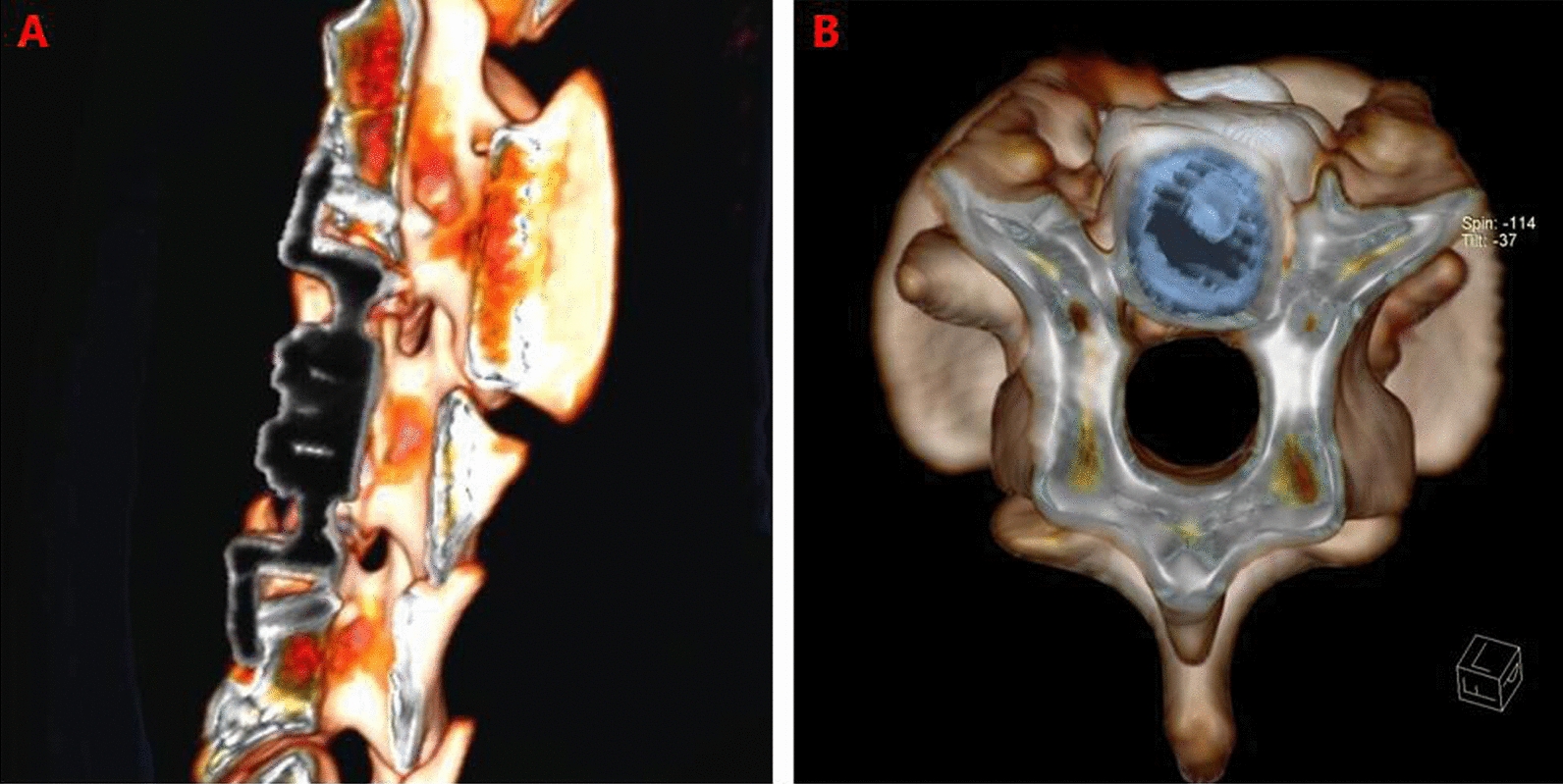
3D CT reconstruction 3 months after surgery. **A**, Sagittal view. **B**, Axial view

**Fig. 7:**
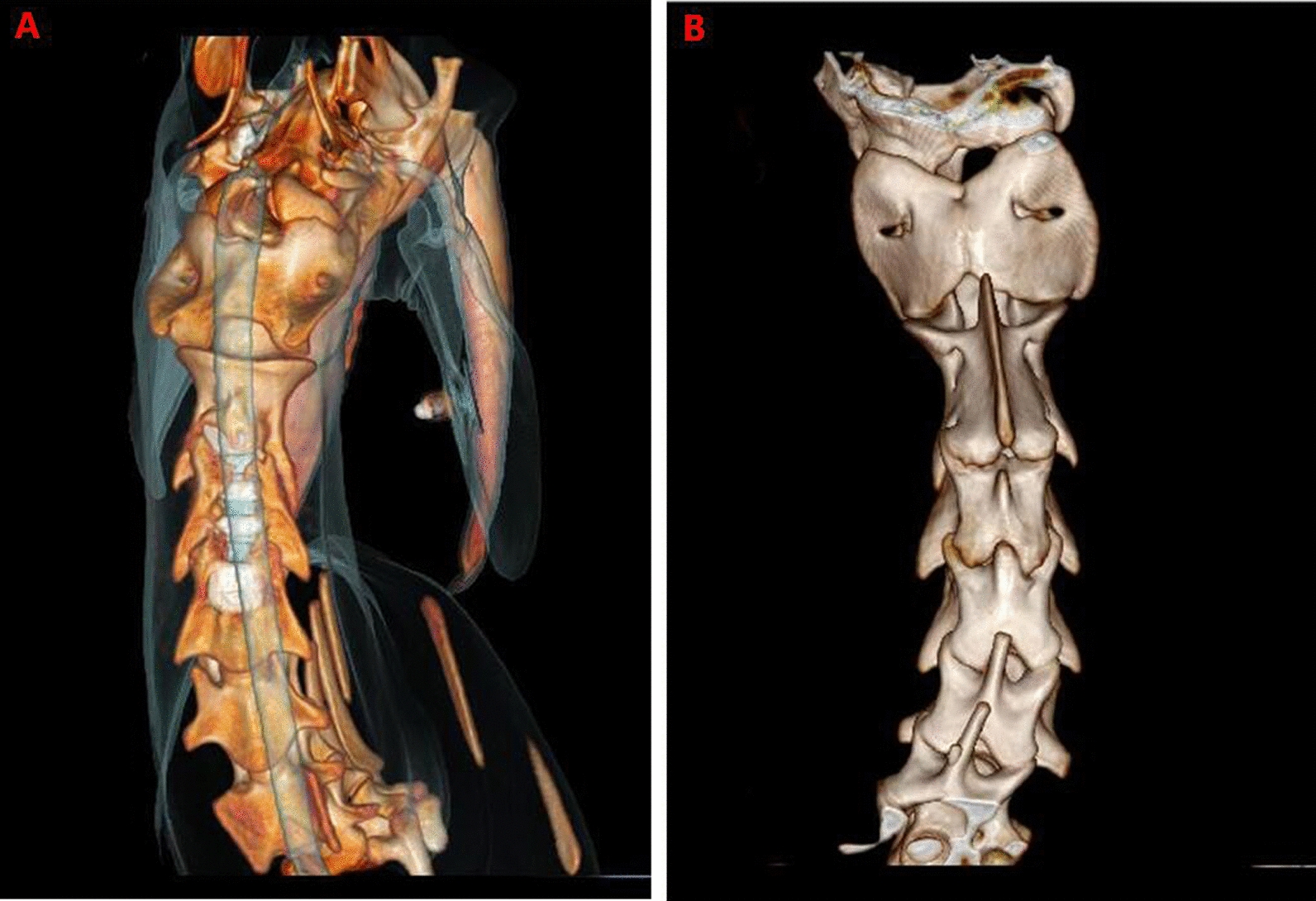
3D CT reconstruction 6 months after surgery. **A**, Front view. **B**, Back view

**Fig. 8:**
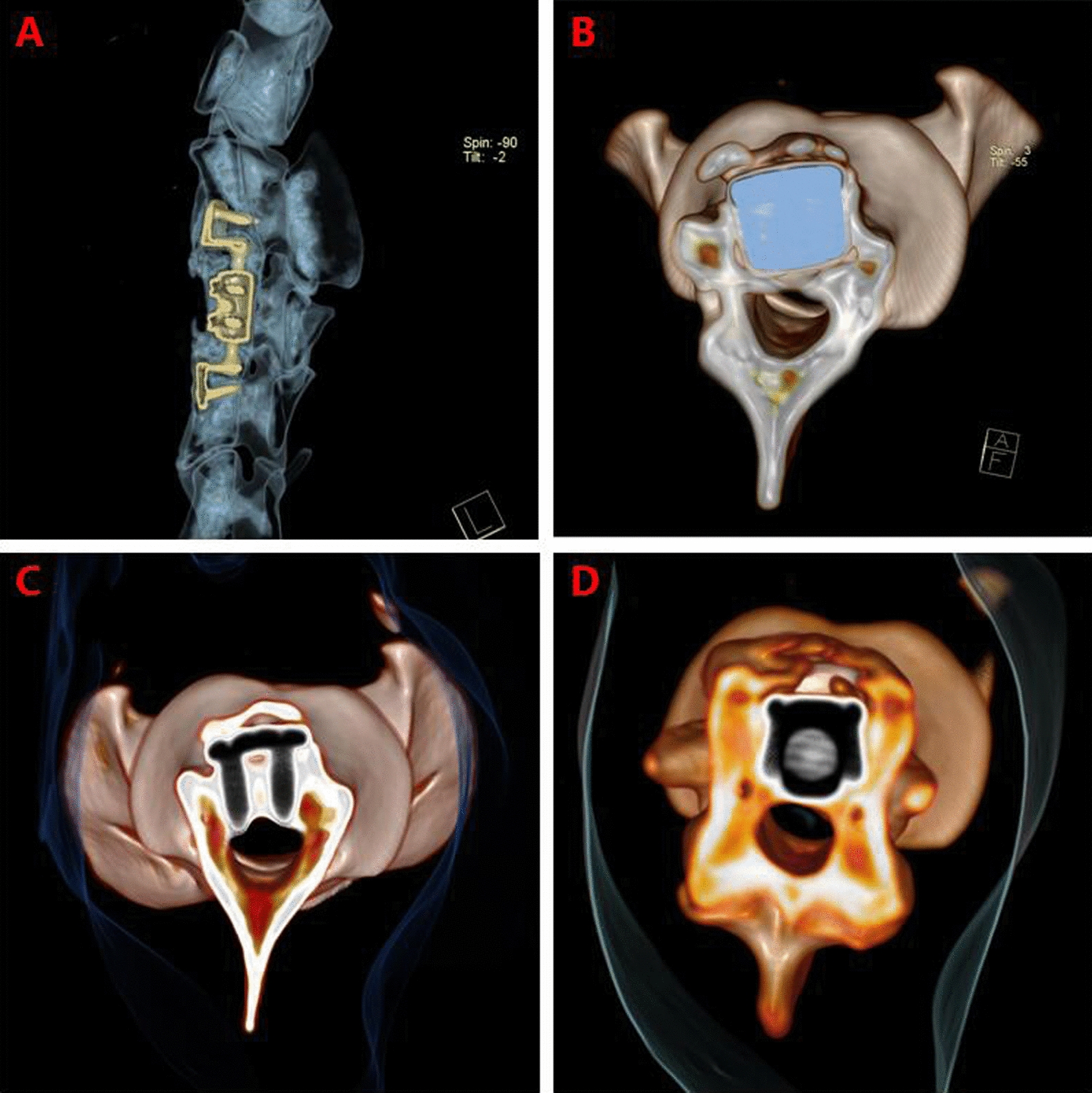
3D CT reconstruction6 months after surgery. **A**, Sagittal view. **B**, **C**, **D**, Axial view

**Fig. 9:**
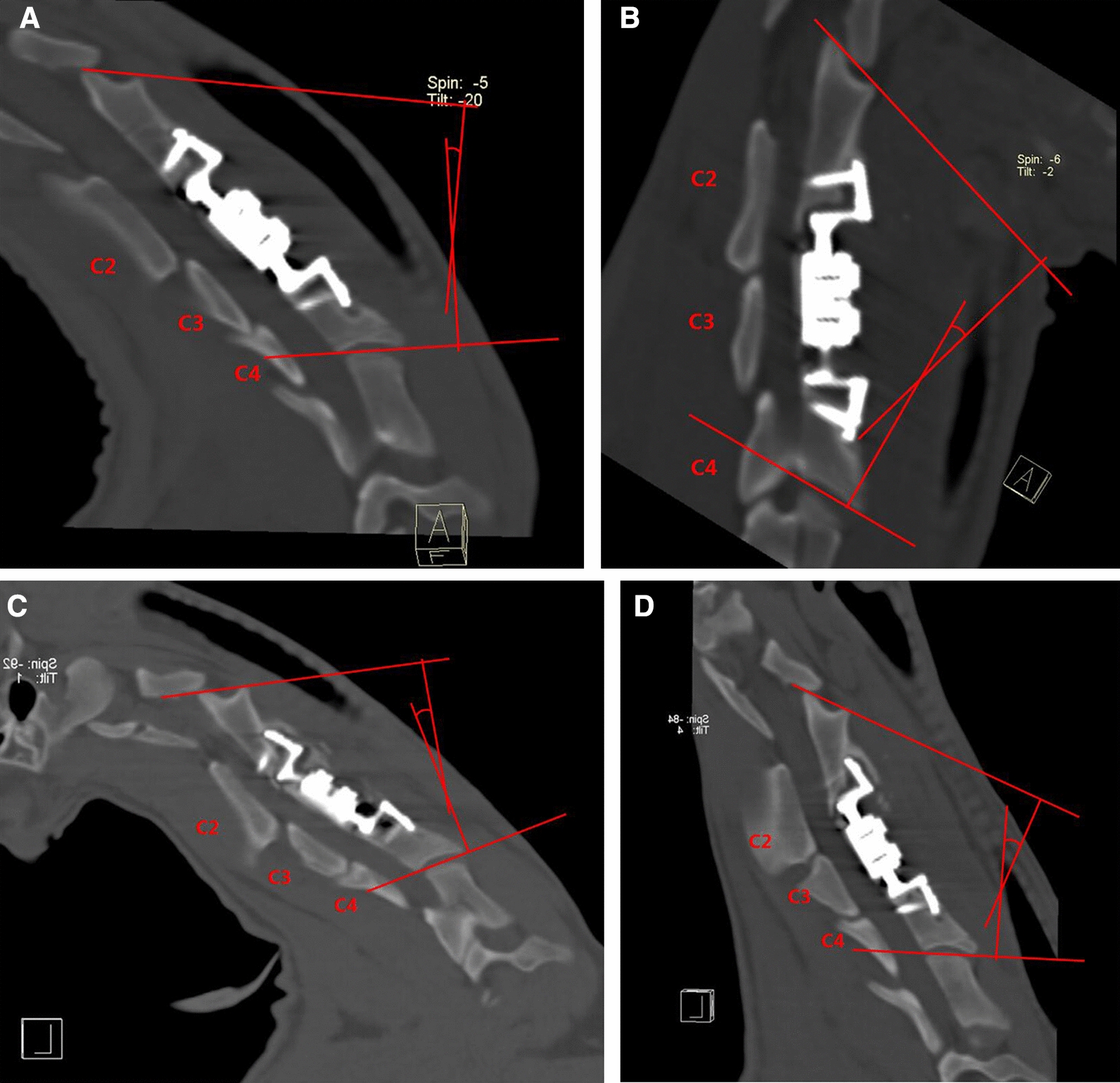
**a** Cobb angle under extension 3 months after surgery. **b** Cobb angle under flexion 3 months after surgery.** c** Cobb angle under extension 6 months after surgery.** d** Cobb angle under flexion 6 months after surgery

3D CT reconstruction indicated that the relationship between the facet joints was stable, no joint dislocation was observed, and only slight heterotopic ossification was noted (Figs. 5, 6, 7, 8). The ROM of the C2-C4 segments during flexion–extension at 3 and 6 months after the operation was 7.8° and 7.3, respectively (Fig. 9 and Table 1).

**Table 1:** Cobb angle of C2-C4 under flexion and extension and ROM of C2-C4 segments under flexion–extension $(\bar{x}\,{\pm}\, {s}, \, ^\circ)$(

	Before surgery	3 months after surgery	6 months after surgery
Flexion	40.2 ± 2.3	17.1 ± 1.5	19.5 ± 2.2
Extension	23.1 ± 2.4	9.2 ± 1.4	12.1 ± 3.3
ROM in flexion–extension	17.1 ± 2.2	7.8 ± 0.5*	7.3 ± 1.4*

^*^A significant difference (*P* < 0.05) was noted between the two groups after surgery and the group before surgery in ROM under flexion–extension

There was no significant difference in the two groups after surgery

**Fig. 10:**
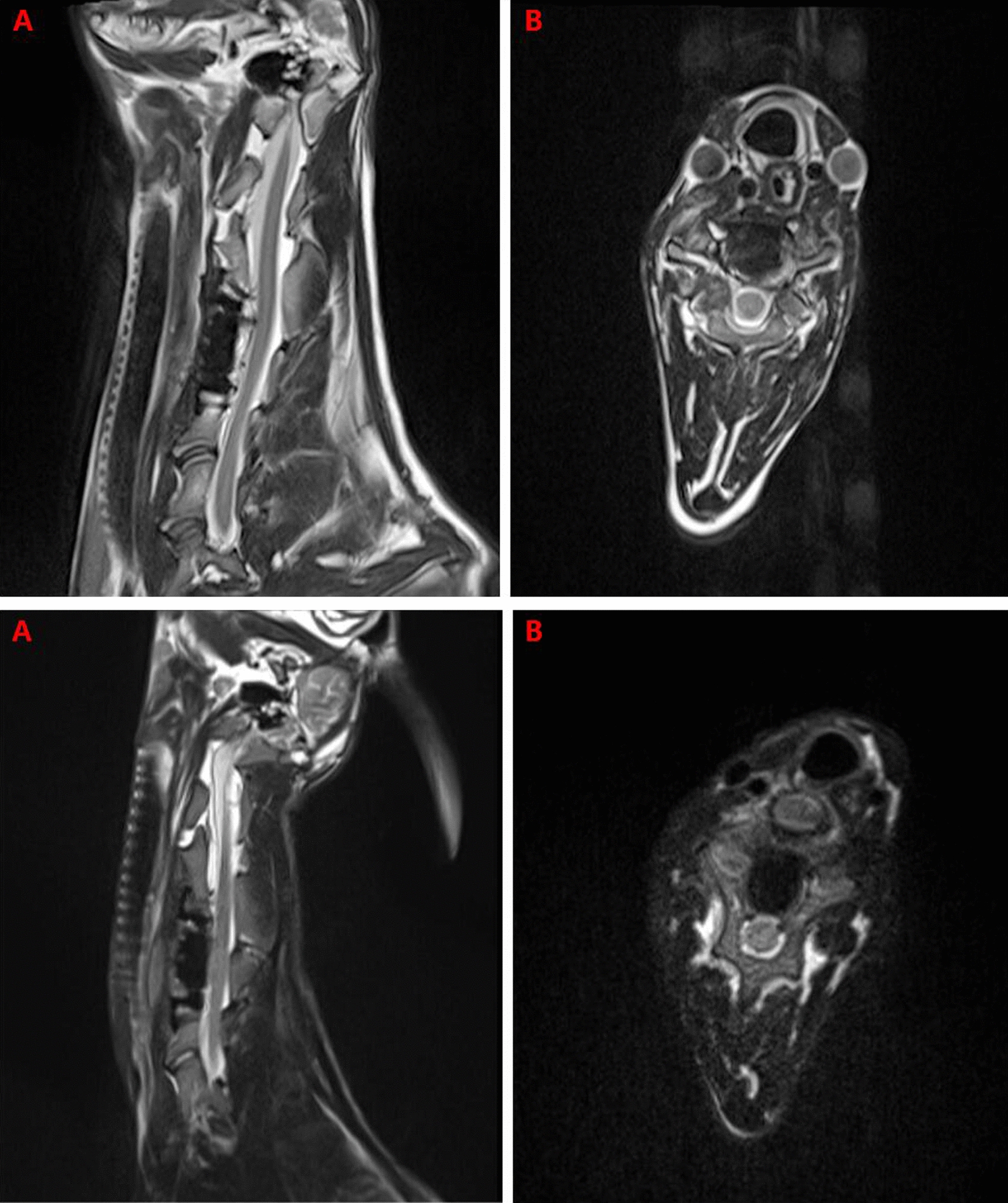
**1** MRI 3 months after surgery. **A**, Sagittal view. **B**, Axial view. **2** MRI 6 months after surgery. **A**, Sagittal view. **B**, Axial view

MRI examination showed no signal abnormalities in the spinal cordor nerve root of the surgical segments, and no degeneration was found in adjacent intervertebral discs (Fig. 10).

### Evaluation of biocompatibility

**Fig. 11:**
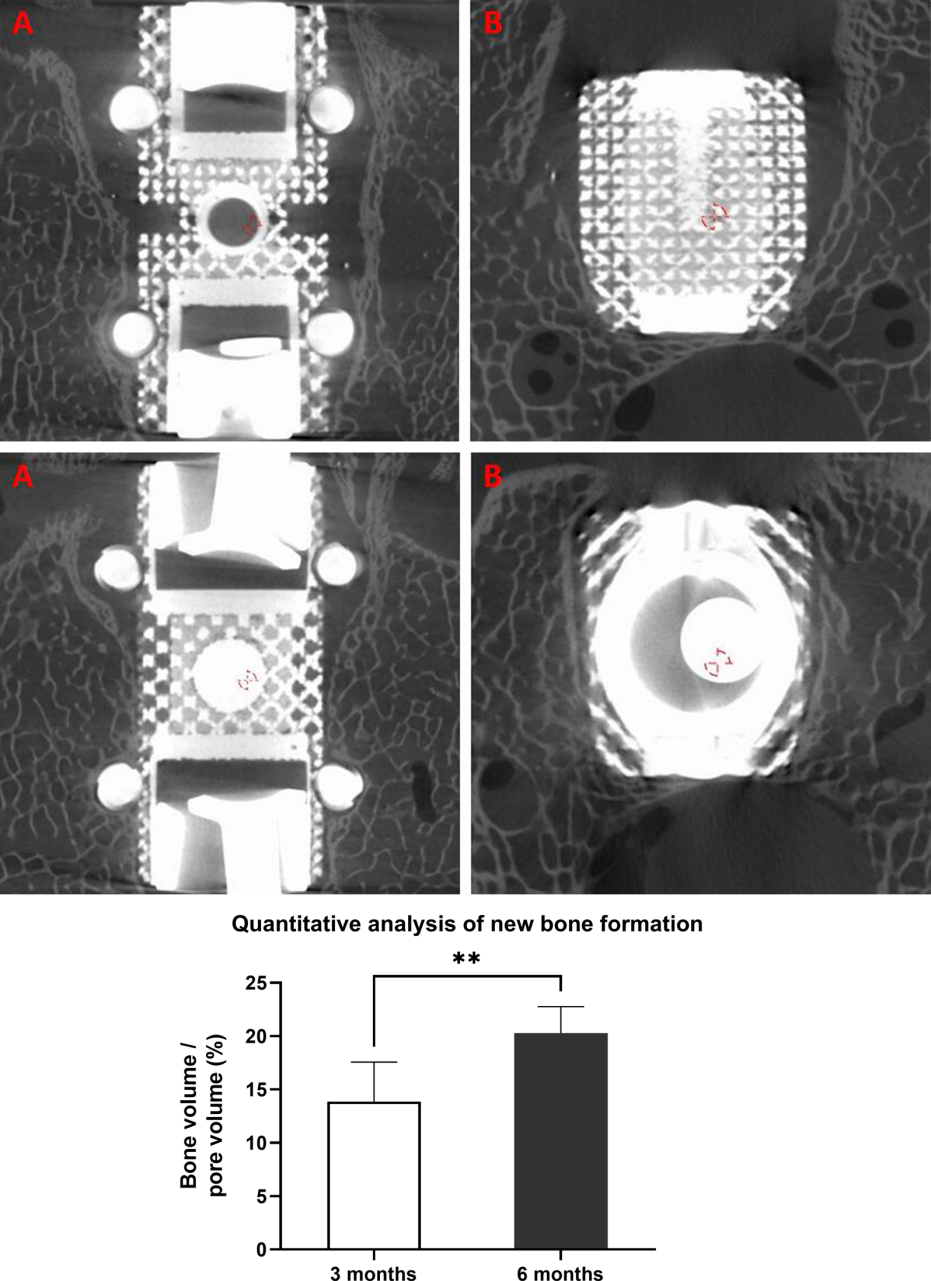
**1** Micro-CT 3 months after surgery. **A**, Coronal scan. **B**, Axial scan. **2** Micro-CT 6 months after surgery. **A**, Coronal scan. **B**, Axial scan. **3** Comparison of BV/PV of 3 months group with 6 months group. The 6 months group has a higher BV/PV (20.3 ± 2.5%) than the 3 months group (13.9 ± 3.7%, *p* < 0.05)

Micro-CT showed new bone tissue around the implant 3 months after the operation. At 6 months, more new bone tissue was observed. We further found that the implant was wrapped in new bone tissue, and new bone tissue had grown into the porous structure of the implant (Fig. 11). The 6 months group has a higher BV/PV (20.3 ± 2.5%) than the 3 months group (13.9 ± 3.7%, *p* < 0.05).

**Fig. 12:**
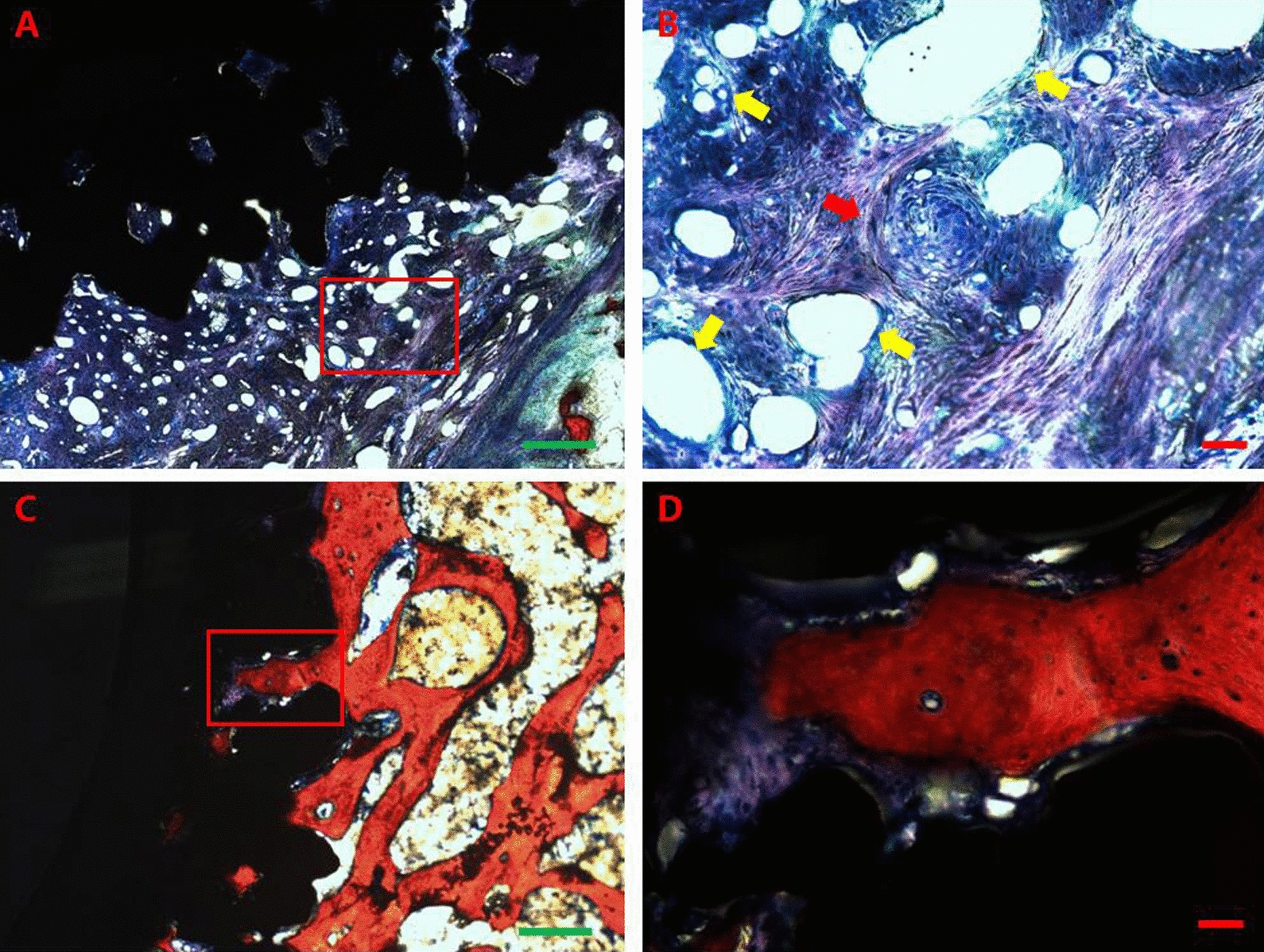
Histological images. **A**, Image of the implant-bone interface at 3 months after surgery (4 ×). A large amount of cartilage tissue was observed at the porous metal vertebrae-bone interface and in the metal pores. **B**, A partial enlargement of the red box shown in image A (20 ×). New blood vessel branching and growth were observed in the cartilage tissue. The nuclei of vascular endothelial cells were visible in the neovascular wall (yellow arrow). An ossification centre was found in the cartilage and had new blood vessels around it (red arrow). Some cartilage tissue appeared reddish, which is a sign of bone mineralization. **C**, Image of the implant-bone interface at 6 months after surgery (4 ×). The cartilage tissue had transformed into mature bone tissue, and a large amount of newly mineralized bone tissue had been generated at the porous metal vertebrae-bone interface and in the metal pores. **D**, A partial enlargement of the red box in image **C** (20 ×). New mineralized bone tissue had generated in the metal pore. Blood vessels distributed within the newly formed bone tissue and cartilage tissue

Histological analysis clearly showed the process of new bone formation after implantation, which involves cartilage tissue formation, angiogenesis, and cartilage mineralization and remodelling. Three months after implantation, a large amount of cartilage tissue could be seen at the porous metal vertebrae-bone interface and in the metal pores. New blood vessel branching and growth (angiogenesis) could be seen in the cartilage tissue, and some cartilage tissue showed signs of transformation into mature bone tissue. The ossification centre, which had new blood vessels around it, was found in the cartilage. The nuclei of vascular endothelial cells were visible in the neovascular wall. Part of the cartilage around the ossification centre appeared reddish, which is a sign of bone mineralization. Six months after implantation, the cartilage tissue had transformed into mature bone tissue, and a large amount of newly mineralized bone tissue had been generated at the porous metal vertebrae-bone interface and in the metal pores. A large number of blood vessels were distributed within the newly formed bone tissue and cartilage tissue (Fig. 12).

## Discussion

ACCF is a common anterior cervical operative method used in spine surgery [10, 11]. Titanium cages have been widely used as implants in ACCF, which replace the resected vertebrae, consequently upholding and maintaining the stability of the cervical vertebrae. However, several of the potential disadvantages of ACCF are postoperative complications induced by titanium mesh implants, such as implant subsidence, which can trigger factors that can cause spinal instability [12]. Stability after corpectomy therefore appears to be very important [13].

In recent years, spine surgeons have been exploring new types of implants to replace titanium mesh. With the rise of metal 3D printing technology, artificial vertebral bodies with metallic trabecular structures can be produced by electron beam melting to acquire perfect results in the experimental stage [14]. In this study, our original purpose was to design a novel 3D-printed porous titanium alloy motion-preservation ACCC to highlight the concept of using 3D printing technology to design porous metal structures. It was our hope that all of these factors would provide reliable spinal stability without the need for autogenous bone grafts, as required by traditional titanium mesh, thus avoiding some of their drawbacks, which give rise to instability of spine. In our study, postoperatively, the necks of the goats could move freely in a normal manner without any external fixation, and the results of the imaging evaluations illustrate that the ACCC was reliable for re-establishing the stability of the anterior cervical vertebrae.

The facet joint supports the load of the posterior vertebral body, and constitutes a three-joint spinal complex together with the intervertebral disc, which transmits the load applied to the spine [15]. A better artificial disc product with a well-restricted ROM should be able to share the majority of the load, thus reducing the pressure on facet joints [16]. In this study, subtotal C3 vertebral resection, partial inferior C2 and superior C4 resection, and total C2/3 and C3/4 intervertebral disc resection were performed. These factors may have had a substantial impact on the stability of the anterior and middle columns of the spine, and the load caused by this kind of instability may be transmitted to the facet joints if the implantation is inappropriate. We were initially very worried that the high level of activity of active goats might result in failure of the device. However, postoperative CT with 3D reconstruction indicated that the facet joints of the goats were orderly and stable and showed no bone hyperplasia or tapering. We concluded that the ACCC was reliable for stable cervical reconstruction in goats.

One obvious issue observed in cervical and lumbar reconstruction is instability, which can lead to other complications, such as adjacent segment degeneration [17]. This kind of degeneration was defined as ‘adjacent segment degeneration’ and ‘adjacent segment disease’ by Hilibrand and Robbins [18]. ‘Adjacent segment degeneration’ describes the radiological changes observed in the segments adjacent to the surgical fusion level [18]. It should be pointed out that this degeneration may not cause clinical symptoms. ‘Adjacent segment disease’, in contrast, refers to newly developed myeleterosis and radiculopathy of segments adjacent to the fusion level [18]. In this study, the ACCC maintained the movement of the C2-C4 segments at 7.8° and 7.3°of in flexion–extension at 3 months and 6 months after surgery, respectively. Moreover, neither ‘adjacent segment disease’ nor ‘adjacent segment degeneration’ was observed. It seems to demonstrate that ACCC was effective in preserving functions related to the motion of the surgical segments and preventing adjacent segment degeneration and disease.

The stability of the implant is based on two aspects that should be evaluated separately. ‘Initial stability’ indicates that mechanical fixation is established between the implant and bone as soon as the implant is installed. This kind of fixation is mechanical rather than biological [19]. However, ‘long-term stability’, which is achieved during the healing stage, takes place at the implant-bone interface as fresh bone tissue forms on the surface of the implant, and this kind of fixation is biological [19]. As time passes, the effects of initial stability decrease, whereas those of long-term stability increase, and this dynamic process of conversion from mechanical fixation to biological fixation constitutes the course of implantation stability [20]. In this study, the design included four screws on the middle part of the ACCC, which provided close and firm contact with bone immediately after implantation, contributing to initial stability and ensuring that bone remodelling occurred at the implant-bone interface.

In addition, a study of surface structures indicated that the osseointegration of metal implants depends on having a porous design that promotes vascularization and bone ingrowth [21]. The precise design of porous structures can be modulated to promote cell adhesion, proliferation and neovascularization, thus facilitating osseointegration [22]. A pore size larger than 400 microns provides good support for vascularisation [22], while a pore size larger than 100 microns promotes ossification [23]. In this study, the ACCC was designed to take into account the fact that microstructure and porosity impact both biocompatibility and osseointegration. Therefore, the diameter of the porous structures was designed to be between 600 and 800 microns. Furthermore, the porous structure was designed to imitate trabeculae. Constructing such porous microstructures with an appropriate pore size ensures good biocompatibility and osseointegration, with the generation of plenty of new cartilage tissue, vessels and bone tissue generated on the surface and inside of the pores.

There are two types of bone development: endochondral and intramembranous ossification [24]. Intramembranous ossification is involved in the formation of the skull [24]. In contrast, the spine, pelvis and limbs develop via endochondral ossification. The formation of cartilage tissue, new vessels and ossification centres around and within the porous implant indicated that the prosthesis had undergone endochondral ossification by three months postoperatively and that this process continued until six months postoperatively. VG staining performed at six months postoperatively showed that cartilage tissue developed into mature bone tissue at the implant-bone interface and inside the metal pores. This process was complex and regular, illustrating that the ACCC promoted bone regeneration and vascularization. Long-term studies have supported the notion that an appropriate material will support cellular adhesion, proliferation and differentiation [25]. Furthermore, the microstructure of implants was considered a determining factor of cellular adhesion and migration and matrix mineralization and to affect angiogenesis [25]. These two points also demonstrate that the new 3D-printed porous titanium alloy motion-preservation ACCC promoted bone regeneration and vascularization not only due to the selection of a titanium alloy material but also because of the microstructural design.

Our team has been trying out new possibilities of motion-preservation devices for a decade. The ACCC in this study is an upgrade of previous product. In this study, we innovatively combined 3D printing technology and porous titanium alloys. The surface structure and pore size were subtly controlled, which were better for vascularization and bone ingrowth. The four screws on the middle part of the ACCC, which provided close and firm contact with bone immediately after implantation, contributing to initial stability and ensuring that bone remodelling occurred at the implant-bone interface. All of these are innovative solutions that we have come up with after various attempts.

## Limitation

Of course, it is important to acknowledge the limitations of this study, namely a short follow-up period. Additionally, the article does not include a comparative analysis with implants used in ACCF. It is mainly because we considered that ROM of the ACCF operative segments was almost zero, resulting in the absence of an ACCF control group. Furthermore, the design of ACCC is based on the movement pattern of the human cervical spine, and when it was implanted into the goat, the ROM of the goat operative segments was significantly reduced, which in turn explains the movement pattern of the goat cervical spine is significantly different from that of humans. All of these limitations need to be improved in subsequent studies.

## Conclusions

A novel motion-preservation ACCC was fabricated using 3D printing technique in which we combined 3D printing technology, a porous metal structure and motion-preservation joints. This combination helped us to achieve nonfusion fixation of the operative segments, thus providing reliable spinal stability that does not require autogenous bone grafting. Imaging evaluations provided direct evidence that the ACCC provided stabilization while preserving the motion of the functional spinal unit. The biocompatibility evaluation showed that the ACCC promoted bone regeneration and vascularization. This study represents a preliminary examination of the ACCC used for ACCF in a goat model. Maybe, this research was not perfect. We hope it will propel further research of motion-preservation devices.

## Data Availability

Not applicable.

## Abbreviations

ACCC: Artificial cervical corpectomy construct
ACCF: Anterior cervical corpectomy and fusion
ASD: Adjacent segment disease
ACDF: Anterior cervical discectomy and fusion
SEM: Scanning electron microscopy
